# Profiles of Perceived Parental Indulgence Among Finnish Emerging Adults: A Replication–Extension Study

**DOI:** 10.3390/bs16101782

**Published:** 2026-09-30

**Authors:** Ming Cui, Carol A. Darling, Hille Janhonen-Abruquah, Mellissa S. Gordon

**Affiliations:** 1 Department of Human Development and Family Science, Florida State University, Tallahassee, FL 32306, USA; cdarling@fsu.edu; 2 School of Applied Educational Science and Teacher Education, University of Eastern Finland, FI-80100 Joensuu, Finland; hille.janhonen-abruquah@uef.fi; 3 Department of Human Development and Family Sciences, University of Delaware, Newark, DE 19711, USA; msgordon@udel.edu

**Keywords:** indulgent parenting, Finnish emerging adults, latent profile analysis

## Abstract

Built upon parenting theories and empirical studies, indulgent parenting has been operationalized as a multidimensional construct assessing material, relational, and behavioral indulgence. Current research has several limitations in that researchers tend to focus on indulgent parenting of younger children, overlook potential cultural variations, and rely heavily on the variable-centered approach. In this study, with a sample of Finnish emerging adults, we aim to fill the gaps in the current literature by using a person-centered approach to (1) replicate parental indulgence profiles reported in a study of American emerging adults by Cui and colleagues in 2018 and to (2) extend this study and consider maternal and paternal indulgence jointly to examine material–relational–behavioral indulgence pattern similarity/dissimilarity between mothers and fathers. With a sample of 306 Finnish emerging adults, results from latent profile analysis (LPA) suggested two-profile solutions for maternal indulgence and for paternal indulgence, separately. The findings of replication suggested differences from the four-profile solutions of maternal indulgence and paternal indulgence reported by American emerging adults in the study by Cui and colleagues. Extending the separate LPA to joint LPA, results supported a four-profile solution, revealing variations in material–relational–behavioral indulgence patterns between mothers and fathers. Implications are also discussed.

## 1. Introduction

In recent decades, the practice of indulgent parenting has become more common among parents (Clarke et al., 2004, 2014). Researchers have begun to pay more attention to such parenting practice and its impact on children. Although parenting theories suggest that indulgent parenting is a negative parenting behavior (e.g., Steinberg & Morris, 2001), the empirical findings are limited and somewhat mixed (e.g., Coccia et al., 2012; Feng et al., 2024; O. F. Garcia et al., 2021; Jiao & Cui, 2024; Roman et al., 2015). Further, current research is limited in several ways, such as the heavy focus on the effect of indulgent parenting on younger children and adolescents while overlooking emerging adult children, the typical use of a variable-centered approach without considering a person-centered approach, and the inadequate attention to potential cultural variations. In this study, we aim to fill the gaps in the current literature with a replication–extension study. Specifically, with a sample of Finnish emerging adults, we aim to use a person-centered approach to *replicate* the findings of indulgent parenting profiles reported in a sample of American emerging adults by Cui et al. (2018). In addition, we aim to *extend* Cui et al.’s separate analyses of maternal and paternal indulgence to examine the multidimensional patterns of indulgence practice between mothers and fathers.

### 1.1. Theoretical Perspective, Definition, and Measurement of Indulgent Parenting

Based on the categorization of responsiveness and demandingness, parenting theories define indulgent parenting as high in parental responsiveness (warmth, affection) and low in parental demandingness (behavioral control, structure) (Baumrind, 1967; Maccoby & Martin, 1983). According to this parenting framework, indulgent parenting was generally believed to bring negative consequences to child development, especially on behavioral outcomes (Baumrind, 1967). However, empirical studies revealed more nuanced findings and suggested more multifaceted implications.

While assessing indulgent parenting under this traditional framework, researchers, including Baumrind (1967, 1971, 1989, 1991), have tended to focus more on the dimension of low demandingness than high responsiveness. Following such practice, many measures of indulgent parenting generally rely on items assessing low behavioral control while minimizing the aspect of high responsiveness. For example, in the commonly used Parenting Styles and Dimensions Questionnaire (PSDQ, Robinson et al., 1995), out of the 15 items on permissive/indulgent parenting dimension, 14 were about lack of behavioral control (e.g., “I find it difficult to discipline our child,” “I threaten our child with punishment more often than actually giving it”), whereas only 1 item tapped on high responsiveness but lacked behavioral specificity (“I spoil our child”).

In the last two decades, building upon the traditional parenting framework while also recognizing the limitations in the measurements, researchers from different groups proposed a multidimensional approach and, with slightly different labelling, operationalized indulgent parenting with three dimensions (e.g., Clarke et al., 2004, 2014; Cui et al., 2019; Fletcher et al., 1999; Kindlon, 2001): material indulgence (e.g., providing children with many material goods), relational indulgence (e.g., being overly protective, overhelping), and behavioral indulgence (e.g., holding low expectations for responsible behaviors). Empirical studies have established the validity and reliability of this multidimensional construct assessing the concept of indulgent parenting. For example, Cui et al. (2019) reported that the Cronbach’s alpha values were between 0.77 and 0.91 across the three dimensions of maternal and paternal indulgence (reliability) and that the construct was highly correlated with other theoretically relevant constructs (concurrent and predictive validity, e.g., the association between maternal indulgence and emerging adults’ self-regulation was −0.38, *p* < 0.01). Bredehoft and Walcheski (2008) produced a comprehensive assessment of the three-dimensional indulgent construct. This multidimensional approach provides more nuanced definitions of the practice of both high responsiveness and low behavioral control.

### 1.2. The State of the Current Literature on Indulgent Parenting

In a series of studies, Baumrind (e.g., Baumrind, 1971, 1989, 1991) suggested that permissive/indulgent parenting was negatively associated with child outcomes. Other studies also reported similar findings, suggesting a negative impact of indulgent parenting on emotional, behavioral, and social outcomes among children and adolescents (Clarke et al., 2014; Chen et al., 2000; Dwairy, 2004; Llorca et al., 2017; Luyckx et al., 2011). For example, a recent study by Feng et al. (2024) reported that indulgent parenting was associated with adolescents’ maladjustment, including depressive symptoms, anxiety, stress, and aggression. In another study, Jiao and Cui (2024) reported that indulgent parenting was related to adolescents’ fear of missing out (FoMO) through lower self-control and self-efficacy. In a meta-analysis of studies on parenting, Li et al. (2019) suggested that a few studies reported an association between indulgent parenting and lower levels of self-control (e.g., Abedini et al., 2012; Patock-Peckham et al., 2001) and self-efficacy (e.g., Aldhafri, 2011; Keshavarz & Mounts, 2017) among adolescents. The results, however, were rather inconsistent, as some studies found no effects (e.g., Jabagchourian et al., 2014; Tam et al., 2012) or positive effects of indulgent parenting (e.g., F. Garcia & Gracia, 2014; Yousaf, 2015). Despite the theoretical proposition of the negative role of indulgent parenting, the mixed findings warrant a further look into the limitations of the current research in indulgent parenting to better understand the practice of indulgence in developmental and social contexts.

One limitation in the current literature is the heavy focus on the effect of indulgent parenting on younger children and adolescents while ignoring the potential lagged effect on emerging adult children. Targeting emerging adult children may inform the current literature of the inconsistent findings. Younger children and adolescents may feel content (e.g., fewer depressive and anxiety symptoms) derived from their desires being met when their parents are giving everything they want (material indulgence), helping them with things they find challenging (relational indulgence), or shielding them from consequences of misbehaviors (relational indulgence). When these children and adolescents become emerging adults, however, the negative consequences of indulgent parenting may become more salient in that these emerging adult children, who have experienced indulgent parenting in their earlier years and may lack the necessary skills to navigate independent living in emerging adulthood, which could lead to well-being problems (e.g., depressive symptoms, anxiety). Both the theory of emerging adulthood (Arnett, 2000) and empirical studies (e.g., Love et al., 2022) suggest the negative role of indulgent parenting in emerging adulthood. The current mixed findings from samples of younger children and adolescents may suggest the need to look into indulgent parenting in emerging adulthood.

Another limitation in the current literature is the lack of attention to cultural variations, which may contribute to the mixed findings. First, studies of indulgent parenting in different countries have yielded inconsistent findings. For example, parental indulgence was reported to be related to adolescents’ emotional problems in a sample of American adolescents (Feng & Cui, 2023) and behavioral maladjustments in a sample from Spain (F. Garcia & Gracia, 2009). However, Wolfradt et al. (2003) reported that indulgent parenting is related to lower levels of anxiety in Germany. Further, very few studies directly compared indulgent parenting practice across cultures. Using a sample of American adolescents and a sample of Chinese adolescents, Feng et al. (2024) reported that relational indulgence appeared to have stronger association with maladjustment among Chinese adolescents as compared to American adolescents, suggesting potential cultural differences. The limited research warrants further investigation with samples from different cultures regarding indulgent parenting practice.

Finally, current research is limited in that most studies on indulgent parenting use a variable-centered approach (e.g., correlation, regression, and structural equation modeling). Because of the different measures used in assessing indulgent parenting, the mixed findings could partially be due to measurement inconsistency. While it is meaningful to examine mean levels and variations in indulgent parenting using a variable-centered approach, it is equally important to investigate the different patterns or profiles of indulgent parenting, which could complement the variable-centered approach and clarify inconsistences in the findings. There are only a few studies in the literature that use a person-centered approach to study indulgent parenting. Cui et al. (2023) used a person-centered approach and reported a four-profile pattern of indulgent parenting. However, this study used a sample of American adolescents. With a sample of 449 American emerging adults, Cui et al. (2018) examined maternal and paternal indulgent parenting and reported a four-profile solution for both maternal indulgence and paternal indulgence. In the present study, with a person-centered approach, we aim to replicate the Cui et al. (2018) study to better understand indulgent parenting with a sample of emerging adults in a different cultural context in Finland.

Further, even though Cui et al.’s (2018) study was one of the first to investigate profiles of indulgent parenting among emerging adults, their study is limited in that they examine the profiles of maternal indulgence and paternal indulgence separately. The separate analyses do not allow an investigation of the similarity and difference in the material–relational–behavioral indulgence pattern between mothers and fathers within each profile. Studies with a variable-centered approach have shown that, for both maternal and paternal indulgence of adolescent and emerging adult children, the mean levels of material indulgence tended to be the highest, followed by relational indulgence, and lastly behavioral indulgence (e.g., Cui et al., 2018, 2019; Feng et al., 2024; Love et al., 2022). Further, studies have shown that these different dimensions of indulgence parenting may have different implications for adolescents’ and emerging adults’ well-being. For example, Feng et al. (2024) suggested that both maternal and paternal behavioral indulgence had the strongest (positive) relationship with adolescents’ emotional and behavioral problems (e.g., depressive symptoms, anxiety, stress, and aggression). Using a variable-centered approach, one study reported that the profile with high maternal and paternal behavioral indulgence and maternal relational indulgence was associated with the lowest levels of adolescents’ psychological well-being (e.g., autonomy, competence, relatedness, self-control, and self-efficacy) (Cui et al., 2023). In the study by Cui et al. (2018), the authors reported that the profile with high maternal material indulgence and the profile with high paternal relational indulgence were more strongly related to emerging adults’ problems (e.g., depressive symptoms, anxiety, emotional dysregulation, alcohol use). Given the findings of these studies across the three dimensions of indulgence and their different implications for emerging adults’ well-being, the second goal of this study is to extend Cui et al.’s (2018) study by combining maternal and paternal indulgence in the same latent profile analysis and investigate the similarity and dissimilarity in the material–relational–behavioral indulgence patterns between mothers and fathers.

### 1.3. The Present Study

In this study, with a sample of Finnish emerging adults, we aim to fill the gaps in the current literature by using a person-centered approach to (1) replicate parental indulgence profiles reported among a sample of American emerging adults (Cui et al., 2018) and (2) extend the indulgence profiles to consider maternal and paternal indulgence jointly to examine material–relational–behavioral indulgence pattern similarity/dissimilarity between mothers and fathers. First, we explore if the profiles of indulgent parenting are the same in the sample of Finnish emerging adults in this study as compared to the profiles from the sample of American emerging adults in the study by Cui et al. (2018). In the Cui et al. (2018) study, a four-profile solution was reported for maternal indulgence: low indulgence (Profile 1), high indulgence (Profile 2), material-focused indulgence (Profile 3), and moderate indulgence (Profile 4). For paternal indulgence, a four-profile solution was also found: low indulgence (Profile 1), high indulgence (Profile 2), moderate high relational indulgence (Profile 3), and moderate-low relational indulgence (Profile 4). Using the same measure of indulgent parenting, the same latent profile analysis (LPA) was used for replication in this study. Next, as the Cui et al. (2018) study examined maternal indulgence and paternal indulgence separately, we aim to extend their study to examine material–relational–behavioral indulgence pattern similarity/dissimilarity between mothers and fathers, which, from a family system perspective (e.g., treating the family as a system with interrelated subsystems, Cox & Paley, 1997), further contributes to the literature.

## 2. Method

### 2.1. Sample and Procedures

The Finnish participants were recruited from introductory courses at two large universities in Finland in 2017. Students in these classes were invited to participate in an online survey, which contained the same measure of indulgent parenting as the one used in the Cui et al. (2018) study. The survey was translated into Finnish, back-translated, discussed within a focus group, then pilot-tested among a group of Finnish students before it was finalized. The survey asked participants about their perceptions of their mothers’ and fathers’ indulgent parenting practice, along with demographic information. Upon completing the survey, each participant received a movie ticket. A total of 306 students participated and completed the online survey. The average age of the participants was 22.86 (*SD* = 4.02); 87.1% were female, 87.7% were freshmen and sophomore, and 68.0% were from two-parent families. All except for two were of Finnish descent, and 15% were from families with incomes under 30K (in EUR).

### 2.2. Measures

Perceived indulgent parenting was assessed with a 30-item measure tapping on three dimensions of indulgent parenting: material, relational, and behavioral indulgence (Cui et al., 2018, 2019). As with the prompt used in the Cui et al. (2018) study, participants were asked to rate indulgent behaviors of their mother (or mother figure) and father (or father figure) with whom they lived most of the time during their childhood and adolescent years. The measure included 10 items for each dimension: *material indulgence* (e.g., “my mother/father gave me all the clothes I wanted,” Cronbach’s alpha *α* = 0.86 for mothers, *α* = 0.88 for fathers; McDonald’s Omega ω = 0.83 for mothers, ω = 0.88 for fathers), *relational indulgence* (e.g., “my mother/father tried to solve problems for me before I even experienced them,” *α* = 0.77 for mothers, *α* = 0.78 for fathers; ω = 0.79 for mothers, ω = 0.78 for fathers), and *behavioral indulgence* (e.g., “my mother/father let me get away without doing work she/he told me to do,” *α* = 0.74 for mothers, *α* = 0.76 for fathers; ω = 0.70 for mothers, ω = 0.72 for fathers). Each item ranged from 1 = *strongly disagree* to 5 = *strongly agree,* with several items being reverse-coded. Scores for the items were summed within each dimension for mothers and fathers separately, yielding six measures (i.e., maternal material indulgence, maternal relational indulgence, maternal behavioral indulgence, and paternal material indulgence, paternal relational indulgence, paternal behavioral indulgence), with a higher score indicating a higher level of parental indulgence. Besides the Cui et al. (2018) study, several other studies have also established the reliability and validity of this measure (e.g., Cui et al., 2019; Feng et al., 2024; Hong et al., 2025).

### 2.3. Analytic Strategy

Latent profile analysis (LPA) was used to explore the underlying profiles/groups of parents with similar practices of parental indulgence (e.g., Lenzenweger et al., 2007; Wolfe, 1970). The goal was to identify latent profiles based on posterior probabilities of profile membership from the measures of indulgent parenting. A series of LPA models were conducted including the baseline models, with model comparisons starting from number of profiles *k* = 2. By convention and as in the Cui et al. (2018) study, the decision of profile selection was made based on the following information criteria (ICs): the Akaike information criterion (AIC; Akaike, 1974), the Bayesian information criterion (BIC; Schwarz, 1978), the sample size-adjusted Bayesian information criterion (ssBIC; Sclove, 1987), entropy (Ramaswamy et al., 1993), and the adjusted Lo–Mendell–Rubin likelihood ratio test (A-LMR LRT; Lo et al., 2001). Further, the parametric bootstrapped likelihood ratio test (BLRT, with 1000 resamples) was added to improve the reliability and robustness of the comparison tests. Specifically, smaller values of AIC, BIC, and ssBIC indicate a better fit of the LPA. A higher value of entropy (ranging from 0 to 1) indicates more distinguishable profiles (i.e., a better profile solution). A significant *p* value of A-LMR LRT and BLRT (*p* < 0.05) suggest that the *k*-profile solution is better than the *k − 1* profile solution. As the criteria do not always agree/converge (because each criterion has its own strengths and limitations), the choice of profile selection may not be straightforward (e.g., McLachlan & Peel, 2000; Nylund et al., 2007; Tofighi & Enders, 2008). In addition to comparing these criteria, consideration is also given to theoretically meaningful profiles as well as sufficient profile sizes. Full information maximum likelihood (FIML) was used to handle missing data. All analyses were performed in Mplus (Muthén & Muthén, 1998–2017).

### 2.4. Comparability with the Cui et al. (2018) Study

In the present study, we made every attempt to replicate the Cui et al. (2018) study. The Cui et al. (2018) study recruited college students from introductory courses from two large universities in the U.S. and reported a final sample size of 449. Of the 449 participants, 89.1% were female. The reason for the imbalanced gender composition in the sample was that the survey was distributed to students from colleges of education, human, and social sciences. In this study, we followed the same approach and recruited participants from two large universities in Finland, and 87.1% were female. The racial and ethnic backgrounds of the participants were 84% white, 12% black, 3% Asian, 1% other, and 68% were non-Hispanic. The participants in the Cui et al. (2018) study were sent an online survey that included assessments of their reports of their parents’ indulgent parenting, along with demographic information. In the present study, we used the same indulgent parenting measure and translated the survey into Finnish. The internal consistency in the Cui et al. (2018) study (i.e., 0.90—mothers and 0.92—fathers for material indulgence, 0.82 s—mother and 0.80 s—father for relational indulgence, and 0.84—mothers and 0.80—fathers for behavioral indulgence) was comparable to what was obtained in this study. Given the multidimensionality of the indulgence measure, in this study, we further reported McDonald’s Omega for establishing internal consistency. The validity of this indulgence measure was also supported by the Cui et al. (2018) study (e.g., concurrent and predictive validity). For the analyses, we used the same LPA with the same selection criteria and selection rationale. We further added a parametric bootstrapped likelihood ratio test (BLRT) to provide additional support for profile selection. The similarity in samples, procedures, measures, and statistical approaches strengthened the replication and extension analyses in this study.

## 3. Results

### 3.1. Replication

LPA was used to explore the latent profiles in the Finnish sample. The results of the criteria for the baseline model with a single profile (*k* = 1) were AIC = 6008.06, BIC = 6030.32, and ssBIC = 6011.29 for maternal indulgence and AIC = 5642.82, BIC = 5664.63, and ssBIC = 5645.60 for paternal indulgence. Table 1 provides the selection criteria from *k* = 2 to *k* = 4 groups. The number of initial stage random starts was set at 40, and the number of final stage optimizations was set at 2. The minimum covariance coverage value was 0.10, and the model estimations all terminated normally. The left section of Table 1 shows results for maternal indulgence. The sample size for LPA for maternal indulgence was 302. The two-profile solution produced the smallest BIC. Further, the significant A-LMR LRT test and BLRT suggested that the two-profile solution was significantly better than the one-profile baseline model. The three-profile solution produced the biggest entropy value. However, the A-LMR was not significant, suggesting that the three-profile solution was not better than the two-profile solution. Further, there was insufficient profile size in one profile (i.e., only three counts) for the three-profile solution. The four-profile solution yielded the smallest AIC and ssBIC; however, the A-LMR and BLRT were not significant, the entropy value was the lowest, and there was insufficient profile size in one profile (e.g., only two counts in one profile). Given these considerations, the two-profile solution for maternal indulgence was selected. It is important to note that in the Cui et al. (2018) study, their choice of the best profile solutions, including both maternal and paternal profiles, also involved similar considerations (e.g., inconsistent indications by AIC, BIC, or ssBIC). The findings were remarkably similar, and as a result, our selection rationale was also very comparable with theirs.

**Figure 1:**
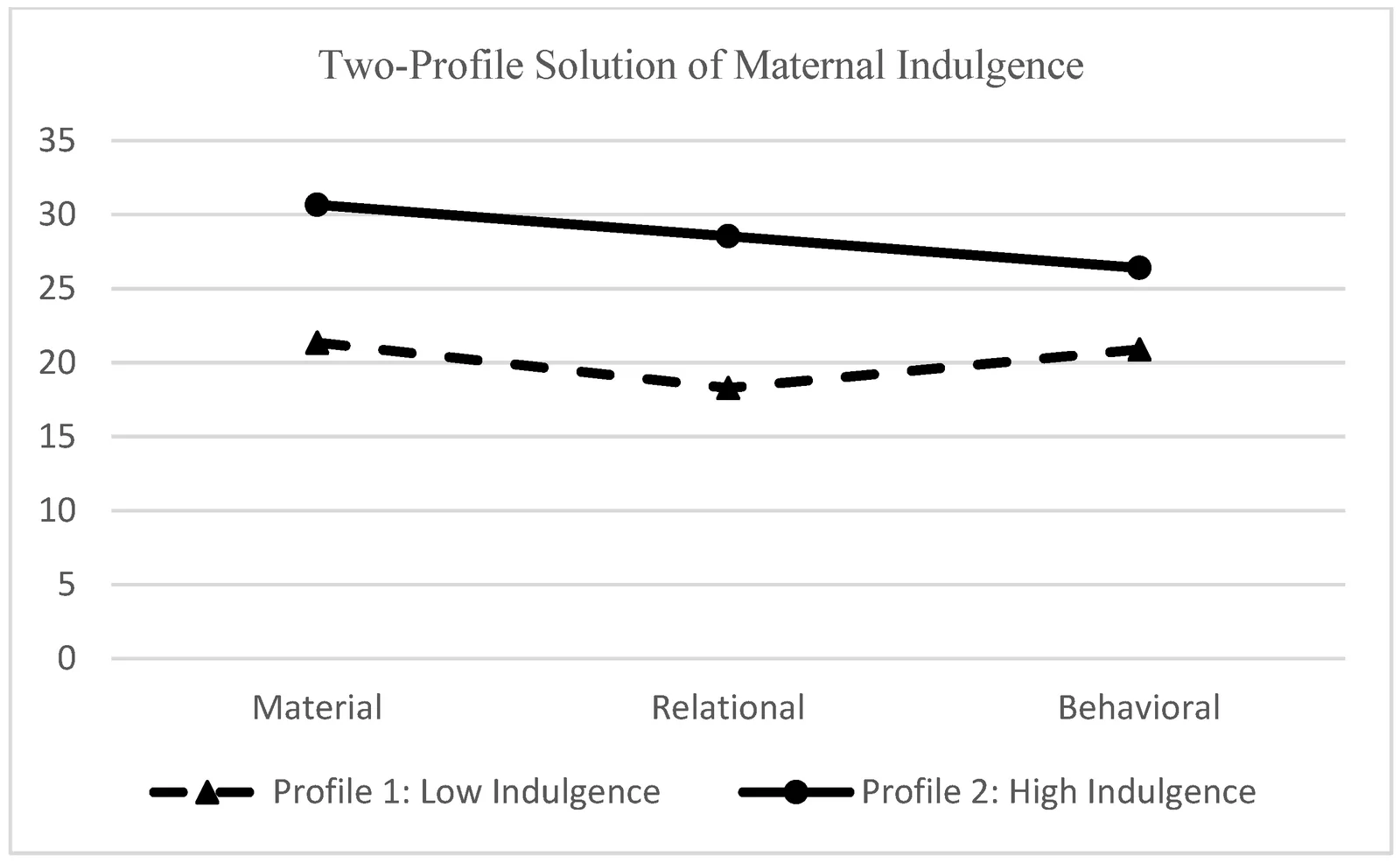
Profiles of maternal indulgence.

Table 2 presents the mean values for each profile of the two-profile solution. The left section shows the results for maternal indulgence. Based on the mean levels, Figure 1 illustrates the two-profile solution for maternal indulgence. Profile 1 (N = 239, 79%) shows lower levels of indulgence across material (M = 21.36), relational (M = 18.29), and behavioral (M = 20.89) dimensions. We labeled this group “Profile 1—Maternal low indulgence.” Profile 2 (N = 63, 21%) shows higher levels of indulgence across the material (M = 30.66), relational (M = 28.54), and behavioral (M = 26.41) dimensions. We labeled this group “Profile 2—Maternal high indulgence.” Equality tests of mean levels between the two profiles suggested that the mean differences in maternal material (χ^2^ (1) = 29.87, *p* < 0.001), relational (χ^2^ (1) = 138.04, *p* < 0.001), and behavioral (χ^2^ (1) = 22.21, *p* < 0.001) indulgence were all significant between the two profiles.

Table 1 also shows the comparisons of the selection criteria from k = 2 to k = 4 groups for paternal indulgence (the right section). Based on the selection criteria, the two-profile solution produced the smallest BIC and significant A-LMR and BLRT. The three-profile solution produced non-significant A-LMR and BLRT, suggesting that the fit was not better than the two-profile solution. Further, there were only two counts in one profile in the three-profile solution. The four-profile solution produced desirable results for most selection criteria, but the small profile size in one profile (i.e., there were only two counts in one profile) made it difficult to form meaningful profiles. In addition, given the disproportional profile size distributions, this small profile may suggest a mathematical artifact (Stanley, 2022). Given these considerations, the two-profile solution was selected as the best fitting model.

**Figure 2:**
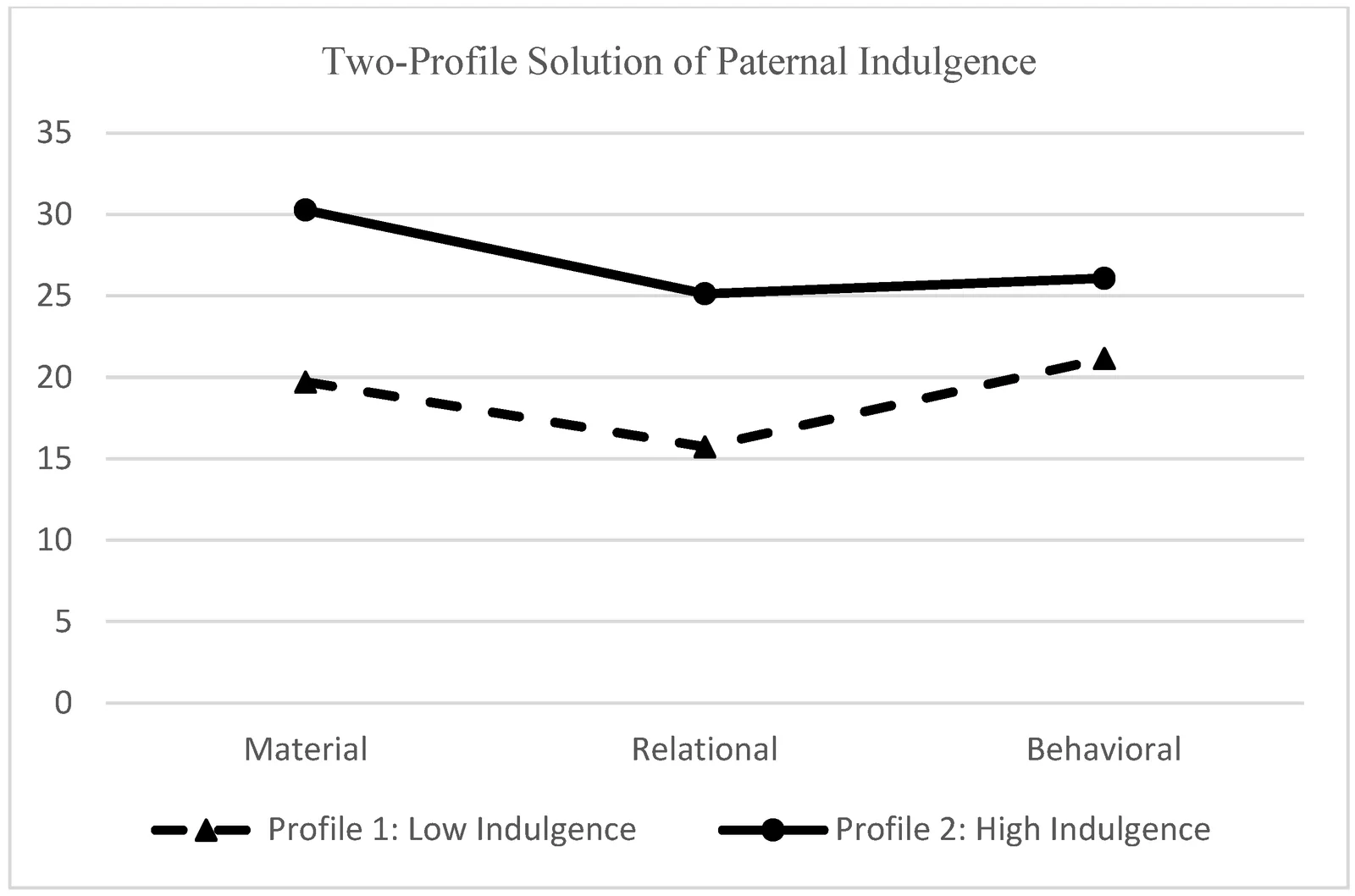
Profiles of paternal indulgence.

Table 2 also presents the mean values for each profile of the two-profile solution for paternal indulgence (the right section). The sample size for LPA for paternal indulgence was 280. Based on the mean levels, Figure 2 illustrates the two-profile solution for paternal indulgence. Profile 1 (N = 201, 72%) shows lower levels of indulgence across the material (M = 19.71), relational (M = 15.74), and behavioral (M = 21.15) dimensions. We labeled this group “Profile 1—Paternal low indulgence.” Profile 2 (N = 79, 28%) shows higher levels of indulgence across the material (M = 30.27), relational (M = 25.13), and behavioral (M = 26.08) dimensions. We labeled this group “Profile 2—Paternal high indulgence.” Equality tests of mean levels between the two profiles suggested that the mean differences in paternal material (χ^2^ (1) = 70.93, *p* < 0.001), relational (χ^2^ (1) = 143.44, *p* < 0.001), and behavioral (χ^2^ (1) = 24.59, *p* < 0.001) indulgence were all significant between the two profiles.

### 3.2. Extension

To extend the previous study, maternal indulgence (i.e., material, relational, behavioral) and paternal indulgence (i.e., material, relational, behavioral) were modeled in the same LPAs to examine material–relational–behavioral indulgence pattern similarity/dissimilarity between mothers and fathers. The results of the criteria for the baseline model with a single profile (*k* = 1) were AIC = 11,650.88, BIC = 11,695.56, and ssBIC = 11,657.50. Table 3 provides the selection criteria from *k* = 2 to k = *5* groups. The number of initial stage random starts was set at 40, and the number of final stage optimizations was set at 2. The minimum covariance coverage value was 0.10, and all the model estimations terminated normally. The sample size for LPA for maternal and paternal indulgence was the full sample of 306. Based on the selection criteria, almost all the criteria converged to suggest that the four-profile solution was the best. Specifically, the four-profile solution produced the smallest BIC and significant A-LMR and BLRT tests. Further, the values of AIC, ssBIC, and entropy were the second smallest. The two-profile solution and the three-profile solution yielded bigger AIC, BIC, ssBIC, and lower entropy values. Even though the five-profile solution produced slightly better values of AIC, ssBIC, and entropy than those in the four-profile solution, the A-LMR test was not significant (though BLRT was), and one profile only had three counts, which prevented the creation of a meaningful profile. Given these considerations, the four-profile solution was selected.

**Figure 3:**
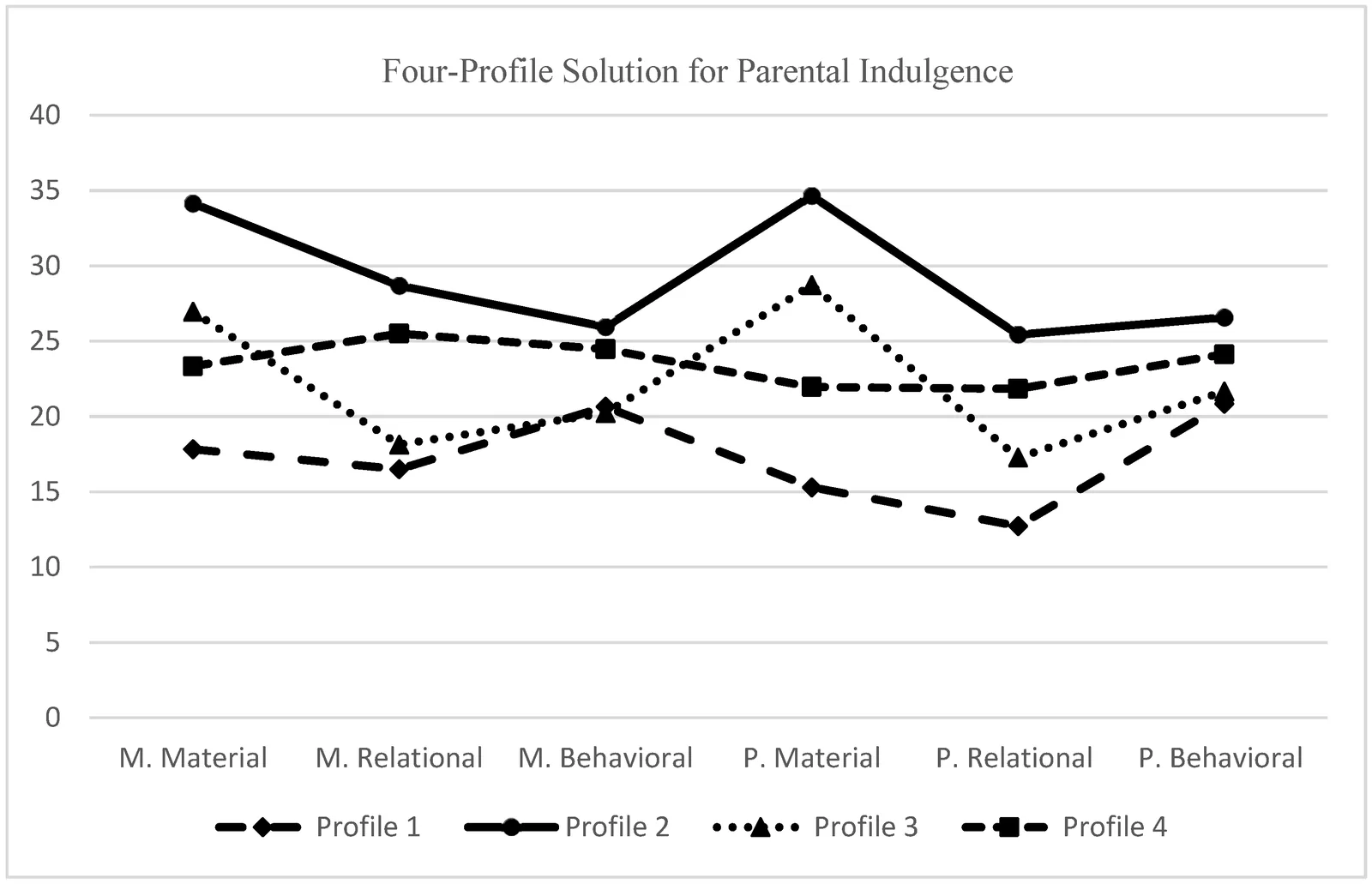
Profiles of parental indulgence. Notes. Profile 1: low indulgence, similar maternal and paternal material–relational–behavioral pattern; Profile 2: high indulgence, similar maternal and paternal material–relational–behavioral pattern; Profile 3: moderate indulgence and material focused, similar maternal and paternal material–relational–behavioral pattern; Profile 4: moderate indulgence, different maternal and paternal material–relational–behavioral pattern.

Table 4 presents the mean values for each profile of the four-profile solution. Figure 3 illustrates the four-profile solution for maternal and paternal indulgence. Profile 1 (N = 120, 39%) shows lower levels of indulgence across the maternal and paternal material, relational, and behavioral dimensions, and the parallel patterns for mothers and fathers suggested a similar material–relational–behavioral pattern between mothers and fathers. We labeled this group “Profile 1—low indulgence, similar maternal and paternal material–relational–behavioral pattern.” Profile 2 (N = 42, 14%) shows highest levels of indulgence across the six indulgence measures and parallel patterns for mothers and fathers. We labeled this group “Profile 2—high indulgence, similar maternal and paternal material–relational–behavioral pattern.” Profile 3 (N = 75, 25%) shows moderate levels of indulgence and parental similarity. We labeled this group “Profile 3—moderate indulgence and material-focused, similar maternal and paternal material–relational–behavioral pattern.” Finally, Profile 4 (N = 69, 22%) shows moderate levels of indulgence but dissimilar maternal and paternal material–relational–behavioral pattern, unlike any other groups. Specifically, mothers in this group showed relatively high levels of relational indulgence as compared to material and behavioral indulgence, whereas fathers showed the opposite pattern. We labeled this group “Profile 4—moderate indulgence, different maternal and paternal material–relational–behavioral pattern.”

To better quantify the profiles established above regarding differences in profiles and between parents, a repeated-measures ANOVA was performed with the four profiles’ membership (i.e., Profiles 1, 2, 3, and 4) as the “between” factor and maternal and paternal indulgence (i.e., material, relational, behavioral) as the “within” (repeated) factor. Table 5 (top portion) summarizes results from post hoc mean comparisons using Bonferroni tests. For comparisons across the profiles, the mean levels of material, relational, and behavioral indulgence were all significantly different across the four profiles (e.g., mean difference between Profile 1 and Profile 2 in material indulgence = −17.66, *p* < 0.001), except for between Profile 1 and Profile 3 and between Profile 2 and Profile 4 on behavioral indulgence. Overall, the findings supported the differentiation among “high,” “moderate,” and “low” levels of indulgence as established and labeled in the profiles.

Regarding material–relational–behavioral pattern similarity/dissimilarity between mothers and fathers, the patterns of the mean levels across the three dimensions of indulgence were compared between mothers and fathers in each profile to quantify the description of material–relational–behavioral pattern similarity or dissimilarity. The results are also shown in Table 5 (bottom portion). For Profile 1, the pattern across maternal and paternal indulgence, as indicated in the sign and significance of the mean differences, was the same (i.e., behavioral being the highest, followed by material, relational being the lowest) with the differences across the three dimensions being consistent across mothers and fathers. To illustrate, for example, the mean difference between material and behavioral indulgence was −2.89 for mothers and −5.64 for fathers, both of which suggested that behavioral indulgence was higher than material indulgence (reflected by the negative differences) and that the differences were significant (*p* < 0.001 for both). Even though the difference between paternal material and relational indulgence (0.45, ns) was not significant, it was in the same direction as that of mothers (1.23, *p* < 0.05). Overall, the findings suggested “similar maternal and paternal material–relational–behavioral patterns” in Profile 1, hence the label. Similarly, in Profile 2, two out of the three comparisons were in the same directions and significant; therefore, we also concluded “similar maternal and paternal material–relational–behavioral patterns.” Profile 3 showed the strongest evidence of “similar maternal and paternal material–relational–behavioral patterns”, as all three comparisons had the same direction and significance. In Profile 4, however, none of the three comparisons showed consistency in either direction and/or significance. As a result, we established this profile as “different maternal and paternal material–relational–behavioral patterns.” Taken together, the comparisons supported the maternal and paternal material–relational–behavioral pattern similarity/dissimilarity descriptions and labels.

## 4. Discussion

The first goal of this study was to replicate parental indulgence profiles reported among American emerging adults with Finnish emerging adults. With a sample of 306 Finnish emerging adults, results from LPA suggested a two-profile solution for maternal indulgence and a similar two-profile solution for paternal indulgence. Regarding indulgence patterns, both profile solutions for maternal and paternal indulgence yielded a low indulgence profile (the bigger profile) and a high indulgence profile (the smaller profile).

This replication of profile analysis of the Cui et al. (2018) study of American emerging adults suggested different profile solutions. While both studies yielded a low indulgence profile and a high indulgence profile, the Cui et al. (2018) study reported four profiles for maternal indulgence and for paternal indulgence. Specifically, in addition to the low indulgence (Profile 1) and high indulgence (Profile 2) profiles, their study also reported two more profiles: a material-focused indulgence profile (Profile 3) and a moderate indulgence profile (Profile 4). Comparing these four profiles in the Cui et al. (2018) study with the U.S. sample, the two-profile solutions in the Finnish sample in this study only produced the high and low profiles. A tentative explanation for this could be that in the diverse cultural context in the U.S. sample (participants were from various racial and ethnic groups), indulgent parenting practices by these American parents could be more varied, whereas the Finnish parents in this study could be more homogenous in their practice of indulgence. As a result, the profile solutions in the U.S. sample may reveal more nuanced differences among these American parents in their indulgent practices, such as those who were particularly high in material indulgence. However, there could also be a methodological effect in that the sample size in the Finnish study (*N* = 306) was smaller than that in the U. S study (*N* = 449), which may limit its power in identifying additional profiles.

The second goal of this study was to extend the indulgence profiles to consider maternal and paternal indulgence jointly to examine material–relational–behavioral pattern similarity or dissimilarity between mothers and fathers. When examining maternal and paternal indulgence separately using the Finnish sample, we observed that both maternal indulgence and paternal indulgence revealed two profiles—one of low indulgence and one of high indulgence. Then, the question becomes the following: when examining maternal and paternal indulgence together, are their “material–relational–behavioral” indulgence patterns similar or different? The answer to this question is important because past research has shown that the levels of the subdimensions of indulgence were different and that each subdimension of indulgence by mothers and fathers may carry different implications for emerging adults’ development (e.g., Cui et al., 2018, 2019; Love et al., 2022).

Results from LPA of maternal and paternal indulgence jointly revealed four profiles of indulgent parenting with patterns of maternal and paternal material–relational–behavioral similarity/dissimilarity: Profile 1 shows low indulgence and similar maternal and paternal material–relational–behavioral patterns; Profile 2 shows high indulgence and similar maternal and paternal material–relational–behavioral patterns; Profile 3 shows moderate indulgence and is material-focused, with similar maternal and paternal material–relational–behavioral patterns; and Profile 4 shows moderate indulgence, with different maternal and paternal material–relational–behavioral patterns. There are several important implications. First, in addition to the low-indulgence and high-indulgence profiles, there were two additional profiles found when maternal and paternal indulgence were examined together. Such findings revealed some variations in indulgent parenting practices with further differentiation of those somewhere in the middle (moderate indulgence). Second, maternal and paternal indulgent practices are generally similar in material–relational–behavioral indulgence patterns. However, despite the general similar pattern of material–relational–behavioral indulgence, there were some families in which mothers and fathers showed different material–relational–behavioral indulgence patterns. In particular, the relatively high maternal relational indulgence stood out. As previous studies have suggested the particularly harmful effects of maternal relational indulgence (Cui et al., 2023) and inconsistent parenting (Shimkowski & Schrodt, 2012), future studies could contribute to the literature by examining how these profile memberships are associated with emerging adult outcomes.

Taken together, the findings of the current study contribute to the current research by using a person-centered approach to replicate the profiles of parental indulgence and explore the patterns of maternal and paternal material–relational–behavioral indulgence similarity/dissimilarity with a sample of Finnish emerging adults. The findings, however, should be viewed in the light of several limitations. First, this study is cross-sectional, making it difficult to argue whether indulgent parenting is a “parent” effect or a “child” effect. With considerable variations among the parent–child dyads, even though parenting theories suggest negative impacts of indulgent parenting on children, it is also likely that the characteristics and needs of children may promote the practice of indulgent parenting (Gagnon et al., 2020). Future studies could benefit from investigating indulgent parenting profiles longitudinally and including information on emerging adults’ well-being to better understand indulgent parenting and its implications. Second, there were a few limitations in the sample. The sample included college students recruited from two universities in Finland; therefore, the findings have limited generalizability. Relatedly, in this sample (as well as the sample from the Cui et al. study), the majority of the participants were female emerging adults. Future studies would benefit from recruiting more gender-balanced samples to examine potential gender differences. In addition to gender, future studies should utilize samples from diverse backgrounds so findings could be generalized to other populations, settings, or situations. Further, the sample size was not as large as the one from the Cui et al. (2018) study, which could have affected the profile solutions when compared with the results from the U.S. sample. Furthermore, this study did not directly compare profiles with those reported in the U.S. study. Future research should recruit samples to perform direct comparisons to strengthen the generalizability and comparability of the findings. Finally, the measures of indulgent parenting came from emerging adults’ retrospective self-reports of perceived parental indulgence, which could be subjective (Love et al., 2022).

Despite its limitations, this study suggests that patterns of indulgent parenting are complex and could differ across cultures and parents. Building upon this study and the Cui et al. (2018) study, more research should be carried out in this area to better inform prevention and interventions programs that aim to improve parenting education and practice, making it more socially and culturally sensitive. For example, in future studies, there could be further replication and extension of findings regarding the more homogenous patterns of indulgence practice in the Finnish sample in this study when compared to the U.S. sample (Cui et al., 2018), which may suggest the feasibility of more generalized programs in Finland. Further, specific programs could be designed to educate mothers and fathers among certain populations in order to effectively coordinate their parenting practices.

## Figures and Tables

**Table 1:** Profile selection for maternal indulgence and paternal indulgence.

Profile Size (N)	Maternal Indulgence			Paternal Indulgence		
	2-Profile	3-Profile	4-Profile	2-Profile	3-Profile	4-Profile
	239/63	227/72/3	155/37/108/2	201/79	192/86/2	151/32/95/2
AIC	**5911.41**	5903.59	5899.96	**5551.54**	5542.40	5527.55
BIC	**5948.51**	5955.54	5966.75	**5587.89**	5593.28	5592.98
ssBIC	**5916.80**	5911.14	5909.66	**5556.18**	5548.89	5535.90
Entropy	**0.75**	0.80	0.69	**0.73**	0.83	0.85
A-LMR (*p*)	**<0.001**	0.21	0.29	**<0.001**	0.15	0.01
BLRT (*p*)	**<0.001**	0.02	0.14	**<0.001**	0.12	<0.001

Notes. AIC = Akaike information criterion, BIC = Bayesian information criterion, ssBIC = sample size-adjusted BIC, and A-LMR = Adjusted Lo–Mendell–Rubin likelihood ratio test. BLRT = bootstrapped likelihood ratio test. The selected solution is noted in bold.

**Table 2:** Mean values of the selected profiles of maternal and paternal indulgence.

Profile Size (N, %)	Maternal Indulgence		Paternal Indulgence	
	Profile 1	Profile 2	Profile 1	Profile 2
	239, 79%	63, 21%	201, 72%	79, 28%
Material	21.36	30.66	19.71	30.27
Relational	18.29	28.54	15.74	25.13
Behavioral	20.89	26.41	21.15	26.08

Notes. Profile 1: low indulgence, Profile 2: high indulgence for both maternal and paternal indulgence.

**Table 3:** Profile selection for maternal and paternal indulgence.

Profile Size (N)	2-Profile	3-Profile	4-Profile	5-Profile
	197/109	117/155/34	120/42/75/69	121/65/39/3/78
AIC	11,401.09	11,351.46	**11,299.05**	11,284.18
BIC	11,471.83	11,448.28	**11,421.93**	11,433.12
ssBIC	11,411.57	11,365.82	**11,317.27**	11,306.26
Entropy	0.75	0.74	**0.75**	0.79
A-LMR (*p*)	<0.001	0.10	**0.05**	0.55
BLRT (*p*)	<0.001	<0.001	**<0.001**	0.01

Notes. AIC = Akaike information criterion, BIC = Bayesian information criterion, ssBIC = sample size adjusted BIC, and A-LMR = Adjusted Lo–Mendell–Rubin likelihood ratio test. BLRT = bootstrapped likelihood ratio test. The selected solution is noted in bold.

**Table 4:** Mean values of the selected profiles of parental indulgence.

Profile Size (N, %)	Profile 1	Profile 2	Profile 3	Profile 4
	120, 39%	42, 14%	75, 25%	69, 22%
Maternal Material	17.82	34.15	26.95	23.33
Maternal Relational	16.50	28.69	18.13	25.51
Maternal Behavioral	20.65	25.94	20.22	24.47
Paternal Material	15.29	34.66	28.72	21.96
Paternal Relational	12.72	25.44	17.28	21.84
Paternal Behavioral	20.85	26.57	21.66	24.13

Notes. Profile 1: low indulgence, similar maternal and paternal material–relational–behavioral pattern; Profile 2: high indulgence, similar maternal and paternal material–relational–behavioral pattern; Profile 3: moderate indulgence and material focused, similar maternal and paternal material–relational–behavioral pattern; Profile 4: moderate indulgence, different maternal and paternal material–relational–behavioral pattern.

**Table 5:** Comparisons among profiles and between maternal and paternal indulgent parenting.

**Comparisons Profiles**	**Material Indulgence**			**Relational Indulgence**			**Behavioral Indulgence**	
Profile 1 vs. Profile 2	−17.66 ***			−11.25 ***			−5.32 ***	
Profile 1 vs. Profile 3	−11.57 ***			−2.01 ***			−0.09	
Profile 1 vs. Profile 4	−5.99 ***			−8.68 ***			−3.75 ***	
Profile 2 vs. Profile 3	6.09 ***			9.24 ***			5.23 ***	
Profile 2 vs. Profile 4	11.67 ***			2.57 ***			1.57	
Profile 3 vs. Profile 4	5.58 ***			−6.67 ***			−3.66 ***	
**Indulgence Dimensions**	**Profile 1**		**Profile 2**		**Profile 3**		**Profile 4**	
	**M**	**P**	**M**	**P**	**M**	**P**	**M**	**P**
Material vs. Relational	1.23 *	0.45	5.43 ***	9.51 ***	9.33 ***	11.86 ***	−2.72 **	−0.55
Material vs. Behavioral	−2.89 ***	−5.64 ***	8.14 ***	8.22 ***	7.15 ***	7.38 ***	−1.62	−2.71 **
Relational vs. Behavioral	−4.13 ***	−6.08 ***	2.71 *	−1.30	−2.18 **	−4.48 ***	1.10	−2.15 *

Notes. M = Maternal, P = Paternal. * *p* < 0.05, ** *p* < 0.01, *** *p* < 0.001.

## Data Availability

The data presented in this study are available on request from the corresponding author. The data are not publicly available due to privacy.

## References

[B1-behavsci-16-01782] Abedini Y., Zamani B. E., Kheradmand A., Rajabizadeh G. (2012). Impacts of mothers’ occupation status and parenting styles on levels of self-control, addiction to computer games, and educational progress of adolescents. Addiction & Health.

[B2-behavsci-16-01782] Akaike H. (1974). A new look at the statistical model identification. IEEE Transactions on Automatic Control.

[B3-behavsci-16-01782] Aldhafri S. (2011). Self-efficacy and physical self-concept as mediators of parenting influence on adolescents’ school and health wellbeing. Journal of Psychology in Africa.

[B4-behavsci-16-01782] Arnett J. J. (2000). Emerging adulthood: A theory of development from the late teens through the twenties. American Psychologist.

[B5-behavsci-16-01782] Baumrind D. (1967). Child care practices anteceding three patterns of preschool behavior. Genetic Psychology Monographs.

[B6-behavsci-16-01782] Baumrind D. (1971). Current patterns of parental authority. Developmental Psychology.

[B7-behavsci-16-01782] Baumrind D., Damon W. (1989). Rearing competent children. Child development today and tomorrow.

[B8-behavsci-16-01782] Baumrind D. (1991). The influence of parenting style on adolescent competence and substance abuse. Journal of Early Adolescence.

[B9-behavsci-16-01782] Bredehoft D., Walcheski M. (2008). Overindulgence: Parental overindulgence assessment scale (1.2).

[B10-behavsci-16-01782] Chen X., Liu M., Li D. (2000). Parental warmth, control, and indulgence and their relations to adjustment in Chinese children: A longitudinal study. Journal of Family Psychology.

[B11-behavsci-16-01782] Clarke J. I., Dawson C., Bredehoft D. (2004). How much is enough?.

[B12-behavsci-16-01782] Clarke J. I., Dawson C., Bredehoft D. (2014). How much is too much?.

[B13-behavsci-16-01782] Coccia C., Darling C. A., Rehm M., Cui M., Sathe S. K. (2012). Adolescent health, stress and life satisfaction: The paradox of indulgent parenting. Stress and Health.

[B14-behavsci-16-01782] Cox M. J., Paley B. (1997). Families as systems. Annual Review of Psychology.

[B15-behavsci-16-01782] Cui M., Darling C. A., Lucier-Greer M., Fincham F. D., May R. W. (2018). Parental indulgence: Profiles and relations to college students’ emotional and behavioral problems. Journal of Child and Family Studies.

[B16-behavsci-16-01782] Cui M., Graber J., Metz A., Darling C. (2019). Parental indulgence, self-regulation, and young adults’ behavioral and emotional problems. Journal of Family Studies.

[B17-behavsci-16-01782] Cui M., Jiao C., Darling C. A. (2023). Patterns of indulgent parenting and adolescents’ psychological development. Family Relations.

[B18-behavsci-16-01782] Dwairy M. (2004). Parenting styles and mental health of Palestinian–Arab adolescents in Israel. Transcultural Psychiatry.

[B19-behavsci-16-01782] Feng Q., Cui M. (2023). Indulgent parenting and the psychological well-being of adolescents and their parents. Children.

[B20-behavsci-16-01782] Feng Q., Yang Y., Cui M. (2024). Indulgent parenting and adolescents’ maladjustments: The roles of cultural context and parental gender. Children.

[B21-behavsci-16-01782] Fletcher A., Steinberg L., Sellers E. (1999). Adolescents’ well-being as a function of perceived interparental consistency. Journal of Marriage and Family.

[B22-behavsci-16-01782] Gagnon R., Garst B., Kouros C. D., Schiffrin H., Cui M. (2020). When overparenting is normal parenting: Examining child disability and overparenting in early adolescence. Journal of Child and Family Studies.

[B23-behavsci-16-01782] Garcia F., Gracia E. (2009). Is always authoritative the optimum parenting style? Evidence from Spanish families. Adolescence.

[B24-behavsci-16-01782] Garcia F., Gracia E., Selin H. (2014). The indulgent parenting style and developmental outcomes in South European and Latin American countries. Parenting across cultures.

[B25-behavsci-16-01782] Garcia O. F., Lopez-Fernandez O., Serra E. (2021). Raising Spanish children with an antisocial tendency: Do we know what the optimal parenting style is?. Journal of Interpersonal Violence.

[B26-behavsci-16-01782] Hong P., Wu H., Mei Q., He J., Cui M. (2025). Spoiling the spoils: Indulgent parenting costs parents’ well-being more when adolescents deny receiving much indulgence. Journal of Happiness Studies.

[B27-behavsci-16-01782] Jabagchourian J. J., Sorkhabi N., Quach W., Strage W. (2014). Parenting styles and practices of Latino parents and Latino fifth graders’ academic, cognitive, social, and behavioral outcomes. Hispanic Journal of Behavioral Sciences.

[B28-behavsci-16-01782] Jiao C., Cui M. (2024). Indulgent parenting, self-control, self-efficacy, and adolescents’ fear of missing out. Current Psychology.

[B29-behavsci-16-01782] Keshavarz S., Mounts N. S. (2017). Perceived parenting style of fathers and Iranian Adolescents’ self-efficacy: The moderating role of gender and education. The Journal of Genetic Psychology.

[B30-behavsci-16-01782] Kindlon D. (2001). Too much of a good thing.

[B31-behavsci-16-01782] Lenzenweger M. F., McLachlan G., Rubin D. R. (2007). Resolving the latent structure of schizophrenia endophenotypes using expectation-maximum-based finite mixture modeling. Journal of Abnormal Psychology.

[B32-behavsci-16-01782] Li J.-B., Willems Y. E., Stok F. M., Dekovic M., Bartels M., Finkenauer C. (2019). Parenting and self-control across early to late adolescence: A three-level meta-analysis. Perspectives on Psychological Science.

[B33-behavsci-16-01782] Llorca A., Richaud M. C., Malonda E. (2017). Parenting, peer relationships, academic self- efficacy, and academic achievement: Direct and mediating effects. Frontiers in Psychology.

[B34-behavsci-16-01782] Lo Y., Mendell N., Rubin D. (2001). Testing the number of components in a normal mixture. Biometrika.

[B35-behavsci-16-01782] Love H., Cui M., Hong P., McWey L. (2022). Parent and child perceptions of indulgent parenting and female emerging adults’ depressive symptoms. Journal of Family Studies.

[B36-behavsci-16-01782] Luyckx K., Tildesley E. A., Soenens B., Andrews J. A., Hampson S. E., Peterson M., Duriez B. (2011). Parenting and trajectories of children’s maladaptive behaviors: A 12-year prospective Community study. Journal of Clinical Child and Adolescent Psychology.

[B37-behavsci-16-01782] Maccoby E. E., Martin J. A., Mussen P. H., Hetherington E. M. (1983). Socialization in the context of the family: Parent-child interaction. Handbook of child psychology: Vol. 4. socialization, personality, and social development.

[B38-behavsci-16-01782] McLachlan G., Peel D. (2000). Finite mixture models.

[B39-behavsci-16-01782] Muthén L. K., Muthén B. O. (1998–2017). Mplus user’s guide.

[B40-behavsci-16-01782] Nylund K. L., Asparouhov T., Muthen B. O. (2007). Deciding on the number of classes in latent class analysis and growth mixture modeling: A Monte Carlo simulation study. Structural Equation Modeling.

[B41-behavsci-16-01782] Patock-Peckham J. A., Cheong J., Balhorn M., Nagoshi C. T. (2001). A social learning perspective: A model of parenting styles, self-regulation, perceived drinking control, and alcohol use and problems. Alcoholism: Clinical and Experimental Research.

[B42-behavsci-16-01782] Ramaswamy V., DeSarbo W. S., Reibstein D. J., Robinson W. T. (1993). An empirical pooling approach for estimating marketing mix elasticities with PIMS data. Marketing Science.

[B43-behavsci-16-01782] Robinson C. C., Barbara Mandleco B., Olsen S. F., Hart C. H. (1995). Authoritative, authoritarian, and permissive parenting practices: Development of a new measure. Psychological Reports. 77.

[B44-behavsci-16-01782] Roman N. V., David E. L., Moyo A., Schilder L., Lacante M., Lens W. (2015). Parenting styles and psychological needs influences on adolescent life goals and aspirations in a South African setting. Journal of Psychology in Africa.

[B45-behavsci-16-01782] Schwarz G. (1978). Estimating the dimension of a model. Annals of Statistics.

[B46-behavsci-16-01782] Sclove S. L. (1987). Application of model-selection criteria to some problems in multivariate analysis. Psychometrika.

[B47-behavsci-16-01782] Shimkowski J. R., Schrodt P. (2012). Coparental communication as a mediator of interparental conflict and young adult children’s mental well-being. Communication Monographs.

[B48-behavsci-16-01782] Stanley L. J., Frey B. B. (2022). Latent profile analysis. The SAGE encyclopedia of research design.

[B49-behavsci-16-01782] Steinberg L., Morris A. S. (2001). Adolescent development. Annual Review of Psychology.

[B50-behavsci-16-01782] Tam C. L., Chong A., Kadirvelu A., Khoo Y. T. (2012). Parenting styles and self-efficacy of adolescents: Malaysian scenario. Global Journal of Human Social Sciences.

[B51-behavsci-16-01782] Tofighi D., Enders C. K., Hancock G. R., Samuelsen K. M. (2008). Identifying the correct number of classes in growth mixture models. Advances in latent variable mixture models.

[B52-behavsci-16-01782] Wolfe J. H. (1970). Pattern clustering by multivariate mixture analysis. Multivariate Behavioral Research.

[B53-behavsci-16-01782] Wolfradt U., Hempel S., Miles J. N. (2003). Perceived parenting styles, depersonalisation, anxiety and coping behaviour in adolescents. Personality and Individual Differences.

[B54-behavsci-16-01782] Yousaf S. (2015). Parenting style and self-efficacy among adolescents. Parenting.

